# Measurement agreement in percent body fat estimates among laboratory and field assessments in college students: Use of equivalence testing

**DOI:** 10.1371/journal.pone.0214029

**Published:** 2019-03-20

**Authors:** Ryan D. Burns, You Fu, Nora Constantino

**Affiliations:** 1 Department of Health, Kinesiology, and Recreation, University of Utah, Salt Lake City, Utah, United States of America; 2 School of Community Health Sciences, University of Nevada Reno, Reno, Nevada, United States of America; Universidad Autonoma de Madrid, SPAIN

## Abstract

The purpose of this study was to examine the agreement in percent body fat estimates among 7 laboratory and field assessments against dual-emission x-ray absorptiometry using equivalence testing. Participants were 437 college students (mean age = 19.2±0.6 years). Dual-emission x-ray absorptiometry was used as the criterion with hydrostatic weighing, skinfold thickness, air displacement plethysmography, near infrared reactance, and three methods of bioelectrical impedance analysis examined as surrogate assessments. Relative agreement was examined using intraclass correlation coefficients. Group level agreement was examined using equivalence testing. Individual-level agreement was assessed using Mean Absolute Percent Error and Bland-Altman Plots. Single measure intraclass correlation coefficient scores ranged from 0.71–0.80. Hydrostatic weighing, skinfold thickness, air displacement plethysmography, and 4-electrode bioelectrical impedance analysis showed statistical equivalence with the criterion using a 10% Equivalence Interval with absolute mean differences ranging from 1.0%-4.9% body fat. Mean Absolute Percent Error ranged from 11.7% using skinfold thickness to 21.9% using Omron (hand-held) bioelectrical impedance analysis. Limits of Agreement were heteroscedastic across the range of mean scores compared to dual-emission x-ray absorptiometry, with greater mean differences observed at higher levels of percent body fat. Hydrostatic weighing, skinfold thickness, air displacement plethysmography, and 4-electrode bioelectrical impedance analysis showed strong evidence for statistical equivalence with dual-emission x-ray absorptiometry in a sample of college students.

## Introduction

Body composition is a health-related fitness domain that correlates with measures of cardiometabolic health [1,2]. Higher levels of fat mass have been associated with increased incidence chronic diseases and higher mortality rates [3,4]. Because of the links to health outcomes, health-related fitness testing batteries include body composition as a primary assessment domain [5,6]. Thus, it is an important public health priority to establish the psychometric characteristics of various body composition assessments.

Throughout the past several decades, there has been numerous research studies exploring the reliability and validity of body composition assessments [7,8]. At the population level, indices such as Body Mass Index (BMI) are widely used because of its ease of use and ease of interpretation [9], however at the individual level BMI may not be the most valid assessment because of its inability to distinguish between fat mass and fat-free mass [10]. Therefore, more direct assessments of body composition are needed. Unfortunately, many studies that examine agreement among body composition assessments only examine a limited number of lab and/or field assessments, making it difficult to determine psychometric characteristics across a range of modalities [11].

Historically, hydrodensitometry (i.e., hydrostatic weighing) has been considered a criterion or reference assessment of body composition and the accuracy of surrogate assessments are often compared to body fat estimates measured using this modality; however recently, multi-compartment models are used as suitable criterion methods [12, 13]. Dual-emission x-ray absorptiometry DXA is often considered a reference measure because of its 3-compartment methodology and high precision and accuracy compared to other 2- and 3-compartment body composition assessments [14, 15]. In this study, DXA was used as the criterion because it uses a 3-compartment model (bone, protein/muscle, and fat) compared to hydrostatic weighing that uses a 2-compartment model (fat-free mass, fat mass).

Many validation studies comparing surrogate assessments to a criterion use a variation of the general linear model (i.e., t-tests, ANOVA) within the data analytic plan to examine group level agreement. However, use of the general linear model approach has limitations, especially as it pertains to decision-making based on obtained *p*-values [16–18]. For example, a study characterized as having a large sample size may find rejecting the null hypothesis (deciding significant differences exist between group means) relatively easy compared to smaller sample size studies [17]. Rejection of the null hypothesis may lead to erroneous decisions, especially when absolute differences are relatively small [18]. Conversely, studies having small sample sizes may erroneously conclude no differences exist between group means based on obtained *p*-values, even when absolute differences are relatively large [18]. Furthermore, failure to reject the null hypothesis of “no difference” does not necessarily provide evidence for “equivalence” [16].

Equivalence testing is an alternative method to assess measurement agreement. Equivalence testing tests the null hypothesis of “non-equivalence”, or presence of effects large enough to be worthwhile. If differences between two hypothetical group means is sufficiently small, the null is rejected and it is concluded that there is evidence for equivalence between two group means [16]. This “reverse null hypothesis” is practically impossible to reject, as random error will always yield some difference between groups means; therefore, for equivalence testing, researchers define Equivalence Intervals. If an observed mean difference 90% Confidence Interval falls within the designated Equivalence Interval, the null hypothesis is rejected and it is concluded that two measurements are statistically equivalent [16]. For health-related fitness assessments, a 10% Equivalence Interval has been used in the past to examine group-level agreement [19]. As stated in Dixon et al. [16] and Saint-Maurice et al. [19], setting Equivalence Intervals is inherently subjective and there is no universal standard, however a 10% Equivalence Interval is relatively conservative and is an interval that has also been recommended by Robinson et al. [20] for model validation.

Although there are numerous studies examining agreement among body composition assessments, the number of assessments examined within each study are usually limited and the statistical methodology used to examine group level absolute agreement has employed a variation of the general linear model testing group mean differences, which unfortunately, as stated previously, has limitations. There has been a paucity of work examining agreement in percent body fat estimates across a variety of lab and field-based assessments of body composition using equivalence testing. Therefore, the purpose of this study was to examine the agreement in percent body fat estimates among 7 laboratory and field assessments against DXA via use of equivalence testing.

## Materials and methods

### Participants

An a priori power analysis was conducted in STATA v15.0 for a paired-sample mean differences test (t-test) with a conservative small effect size, a paired correlation of *r* = 0.50, and a two-sided alpha level of 0.05; in order to achieve at least 80% statistical power, 199 students would need to be recruited. Participants were a convenience sample of college students (Mean age = 19.2 ± 0.6 years; *N* = 437; 301 females, 136 males) recruited from a research university in the western U.S. All participants were enrolled in an exercise science lab course offered during the Fall, Spring, or Summer semesters. All participants were free from physical injury or any psychological condition that would have precluded them from participating in health-related fitness testing. No students were taking diuretics or any medication that could have confounded body composition assessment. There were no exclusion criteria other than the students had to be enrolled in the exercise science laboratory section. All participant data were de-identified. Research reported in the paper was undertaken in compliance with the Helsinki Declaration.

### Assessments

#### Hydrostatic weighing

Hydrodensitometry (i.e., hydrostatic weighing) was a surrogate lab assessment of body composition. The students entered a stainless-steel weighing tank, were instructed to sit on an underwater swing attached to a scale, and subsequently instructed to expel all air from their lungs. Measurements lasted 3–5 seconds. The test was repeated several times (≤ 5 depending on the participant) in order to obtain a stable underwater weight with the body fully submerged. Body volume was calculated as underwater weight divided by water density following a correction for estimated residual lung volume, which was estimated as a sex-specific proportion of spirometry-measured vital capacity (0.24 for males, 0.28 for females) [21]. Body density was calculated by dividing body mass by body volume. Body density was then converted to percent body fat using the Siri equation [22]. Hydrostatic weighing was found to be a reliable measure of body density with reliability coefficient scores > 0.99 [23].

#### Skinfolds thickness

Seven-site skinfolds thickness assessment was a surrogate field assessment of body composition. Skinfold sites for females and males included the chest, midaxillary, triceps, subscapular, abdomen, suprailliac, and thigh. Skinfold measurements were collected using a Lange Skinfold Caliper (Lange; Ann Arbor, MI, USA) on the right side of the body. Each site was measured twice in a rotating order. If the two measurements differed by more than 2mm, a third measurement was taken. Body density was estimated using the skinfold sum using validated prediction algorithm [24–26], and test-retest reliability of the method has been established [25]. Percent body fat was then estimated using the Siri equation [22]. Trained graduate students performed the skinfolds thickness measurements.

#### Air displacement plethysmography

Air Displacement Plethysmography (ADP) was a surrogate lab assessment of body composition. For ADP, body composition was assessed using BOD POD (COSMED; Concord, CA, USA) and the associated standardized procedures to measure body volume [27]. Calibration of the BOD POD was performed daily. Students were instructed to wear tight fitting clothing and a cap to attenuate potential for measurement error. Once a participant was seated inside the BOD POD chamber, two body volume measurements were taken. Using body weight and volume measurements, body density was calculated and converted to percent body fat using the Siri equation [22]. ADP has been shown to be a reliable assessment of body density with reliability coefficients > 0.99 [28].

#### Near infrared reactance

Near Infrared Reactance (IR) was a surrogate field assessment of body composition. The students’ sex, weight, height and age were entered into the IR device (Futrex-6100 A/ZL, Futrex Inc.; Gaithersburg, MD, USA), which was then was zero-adjusted according to the manufacturer’s instructions. Each student sat with their dominant arm relaxed in on an examination table while the light wand of the IR device was placed on the belly of the bicep at the mid-point between the elbow and the acromion process. Readings were determined using infrared light that penetrated 1 cm into the tissue. Scans were made over a range of wavelengths from 700–1,100 nm and the average of 6 optical density readings were used to obtained percent body fat. A light shield was used to block out any surrounding light which could affect the measurement. IR has shown to be a reliable assessment of percent body fat with reliability coefficients > 0.95 [29].

#### Omron bioelectrical impedance analysis

Two-electrode hand-to-hand Bioelectric Impendence Analysis (BIA) was administered using the Omron handheld device. Omron BIA was a surrogate field assessment of body composition. The students’ height, weight, age, and sex were entered into a handheld OMRON Body Fat Analyzer (Model HBF-306; Lake Forest, IL, USA). The students then held the analyzer with arms extended, parallel to the floor until the device displayed the student’s body fat percentage. Stability of hand-held Omron BIA devices has been established in college students with test-retest reliability coefficients > 0.97 [30].

#### Tanita bioelectrical impedance analysis

Tanita BIA was a surrogate field assessment of body composition. Two-electrode foot-to-foot BIA was administered using a Tanita Scale plus BIA (Model BF-556, Tanita; Arlington Heights, IL, USA). The students’ height, weight, age, and sex were entered into the scale. The student’s stood on the scale with shoes removed until a reading was obtained. Height (in meters), weight (in kilograms), and percent body fat were obtained using the Tanita Scale plus BIA. Tanita scales have shown to have excellent test-retest reliability with coefficients > 0.99 [31].

#### Valhalla bioelectrical impedance analysis

Four-electrode Valhalla BIA was a surrogate lab assessment of body composition using the RJL Quantum II, which is a four terminal single frequency (800 mA at 50 kHz) impedance plethysmograph (Valhalla Scientific Model 1990B; Clinton Twp., MI, USA). The calibration procedure uses an internal calibration system. Students wore light clothing and were barefoot (or removed the shoe and sock from the right foot). Students reclined in a supine position on an examination table with arms adjacent to the body, palms flat against the table, and legs adjacent to each other but not touching. Four surface self-adhesive spot electrodes were placed on the dorsal surface of the right hand and on the dorsal surface of the right foot. Prior to placement of electrodes the skin was wiped with alcohol at the 4 locations for electrode placement. Resistance and reactance values were determined on the right side of the body. Two trials were performed and recorded for each subject. The mean of these two trials was used in the calculation to estimate percent body fat. Valhalla BIA has produced consistent test-retest reactance values with differences less than 1% [32].

#### Dual-emission x-ray absorptiometry

DXA was the criterion lab assessment for body composition. DXA (Hologic Discovery W, software version 12.1, Hologic Inc.; Bedford, MA, USA) provides accurate and precise measurements of body bone mineral content and total fat mass with precision scores < 2% [33]. Body composition was divided into bone mass and soft tissue mass. Soft tissue mass was further divided into fat mass and fat-free mass. Percent body fat was calculated by dividing the fat mass by total body mass. DXA procedures were carried out via a trained and certified administrator within a private screening room.

#### Estimated VO_2 Peak_

The sub-maximal Astrand-Ryhming cycle ergometer test was used to estimate VO_2Peak_ [34]. Participants completed the standard protocol for the sub-maximal Astrand-Ryhming cycle ergometer test. The Astrand-Ryhming sub-maximal cycle ergometer protocol was performed in six-minutes [34]. Heart rate was recorded using Polar Heart Rate Monitors (Polar Electro, Lake Success, NY, USA). The procedures for acquiring estimated VO_2 Peak_ were aligned with standard procedures using the heart rate extrapolation method. Maximum heart rate was estimated using the equation 220 –age. Before testing, participants rested quietly for 5 minutes so that heart rate could lower to approximate resting levels.

### Procedures

Assessments of body composition were administered during lab sections of an exercise science course. The body composition field assessments (skinfolds thickness, two-electrode BIA, IR) were collected first and the lab measures (hydrostatic weighing, DXA, ADP, 4-electrode BIA) were collected second. Testing order was assigned randomly for both lab and field assessments. Students were instructed to follow specific guidelines before reporting to the lab for body composition assessment. These guidelines included: 1.) avoiding large meals at least 2 hours prior to testing; 2.) avoiding vigorous physical activity or exercise at least 12 hours prior to testing; 3.) avoiding alcohol at least 48 hours prior to testing; 4.) consumption of liquids should be limited to 2 glasses of water at least 2 hours prior to testing; 5.) emptying bladder immediately prior to testing. Adherence to these guidelines were verbally confirmed by the student prior to testing. Aerobic capacity assessment took place during a separate lab section.

### Statistical analysis

Data were screened for outliers using boxplots and z-scores. Seventeen cases were dropped because of extreme scores that were identified using boxplots that had a z-score > + 3.0z (3.7% of sample). Differences between sexes on the descriptive variables were analyzed using independent t-tests, assuming unequal variances because of the discordance in group sample sizes. Effect sizes were calculated using Cohen’s delta (*d*), where *d* < 0.20 indicating a small effect, *d* = 0.50 indicating a medium effect, and *d* > 0.80 indicating a large effect [35]. Relative agreement in assessment percent body fat estimates with DXA was analyzed using Intraclass Correlation Coefficients (ICCs) via two-way mixed models. ICC scores were computed for each surrogate assessment against DXA. Agreement was considered poor if ICC < 0.50, moderate if ICC = 0.50–0.74, good if ICC = 0.75–0.90, and excellent if ICC > 0.90 [36].

Group level agreement was examined using equivalence testing. Equivalence testing was employed to test the null hypothesis that there was no equivalence (non-equivalence) between the criterion (DXA) and surrogate assessments of percent body fat. A ±10% Equivalence Interval was employed to test the null hypothesis using the confidence interval method at an alpha level of 0.05 or 5%. As described in Dixon et al. [16], if an alpha = 5% test of equivalence is employed, a 90% Confidence Interval needs to be calculated for the difference in means. Therefore, mean differences between percent body fat measured using DXA and each surrogate assessment of percent body fat were reported along with 90% Confidence Intervals. If a calculated mean difference 90% Confidence Interval fell entirely within the Equivalence Interval (i.e., no values outside the Equivalence Interval), the null hypothesis was rejected and it was deemed that two observed assessments of body composition were statistically equivalent. Secondary analyses tested statistical equivalence against a 5% and a 15% Equivalence Interval.

Agreement at the individual level was assessed using the mean absolute percent error (MAPE). MAPE was calculated, using DXA as the criterion, for all assessments of body composition. Finally, Bland-Altman Plots were also used to examine individual-level agreement between each surrogate assessment with DXA using STATA’s “batplot” command [37]. The 95% limits of Agreement were adjusted for heteroscedasticity to show the variability in mean differences across the range of scores [38]. This methodology can provide more precise information compared to conventional Bland-Altman Plots. The adjusted 95% Limits of Agreement were calculated by regressing the mean differences on to the means. Systematic bias was assessed correlating the residual (differences) against mean scores. All analyses were conducted using STATA v.15.0 statistical software package (College Station, Texas, USA).

## Results

Descriptive statistics are reported in Table 1. Males had significantly higher BMI compared to girls (mean difference = 3.3 kg/m^2^, *p* < 0.001, *d* = 0.78), however girls had higher percent body fat, measured using DXA (mean difference = 7.4%, *p* < 0.001, *d* = 1.01). Table 2 communicates the single measure and average ICC scores using two-way mixed methodology. Single measure ICC scores ranged from moderate-to-good and average ICC scores ranged from good-to-excellent across assessments. The assessment with the highest single measure ICC score with DXA was ADP and the assessment with the highest average ICC score with DXA was skinfolds thickness.

**Table 1:** Descriptive statistics (means and standard deviations).

	Female (*n* = 301)	Male (*n* = 136)	Total Sample (*N* = 437)
Age (years)	19.5 (0.6)	18.9 (0.7)	19.2 (0.6)
Body Mass Index (kg/m^2^)	24.0 (4.0)	**27.3**^**†**^ **(4.3)**	24.8 (4.2)
Estimated VO_2 Peak_ (ml/kg/min)	41.8 (11.2)	42.4 (12.1)	42.0 (12.0)
DXA (% Body Fat)	**28.2%**^**†**^ **(6.9%)**	20.8% (7.0%)	27.0% (7.6%)

*Note*: DXA stands for dual emission x-ray absorptiometry; Bold and † denotes statistical differences between sexes, *p* < 0.05.

**Table 2:** Intraclass correlation coefficients against dual emission X-ray absorptiometry (N = 437; ICC with 95% confidence Intervals).

Assessment	ICC (3,1)	ICC (3,2)
Hydrostatic Weighing	0.80 (0.76–0.83)	0.89 (0.86–0.91)
Skinfold Thickness	0.73 (0.66–0.79)	0.93 (0.90–0.94)
ADP	0.84 (0.81–0.87)	0.92 (0.90–0.93)
IR	0.79 (0.75–0.82)	0.89 (0.87–0.91)
Omron BIA	0.77 (0.73–0.81)	0.88 (0.86–0.90)
Tanita BIA	0.75 (0.71–0.79)	0.84 (0.81–0.88)
Valhalla BIA	0.77 (0.73–0.81)	0.86 (0.83–0.88)

*Note*: ICC stands for Intraclass Correlation Coefficient; (3,1) stands for two-way mixed single measure agreement; (3,2) stands for two-way mixed average agreement; ADP stands for air displacement plethysmography; IR stands for near infrared reactance; BIA stands for bioelectrical impedance analysis.

**Fig 1:**
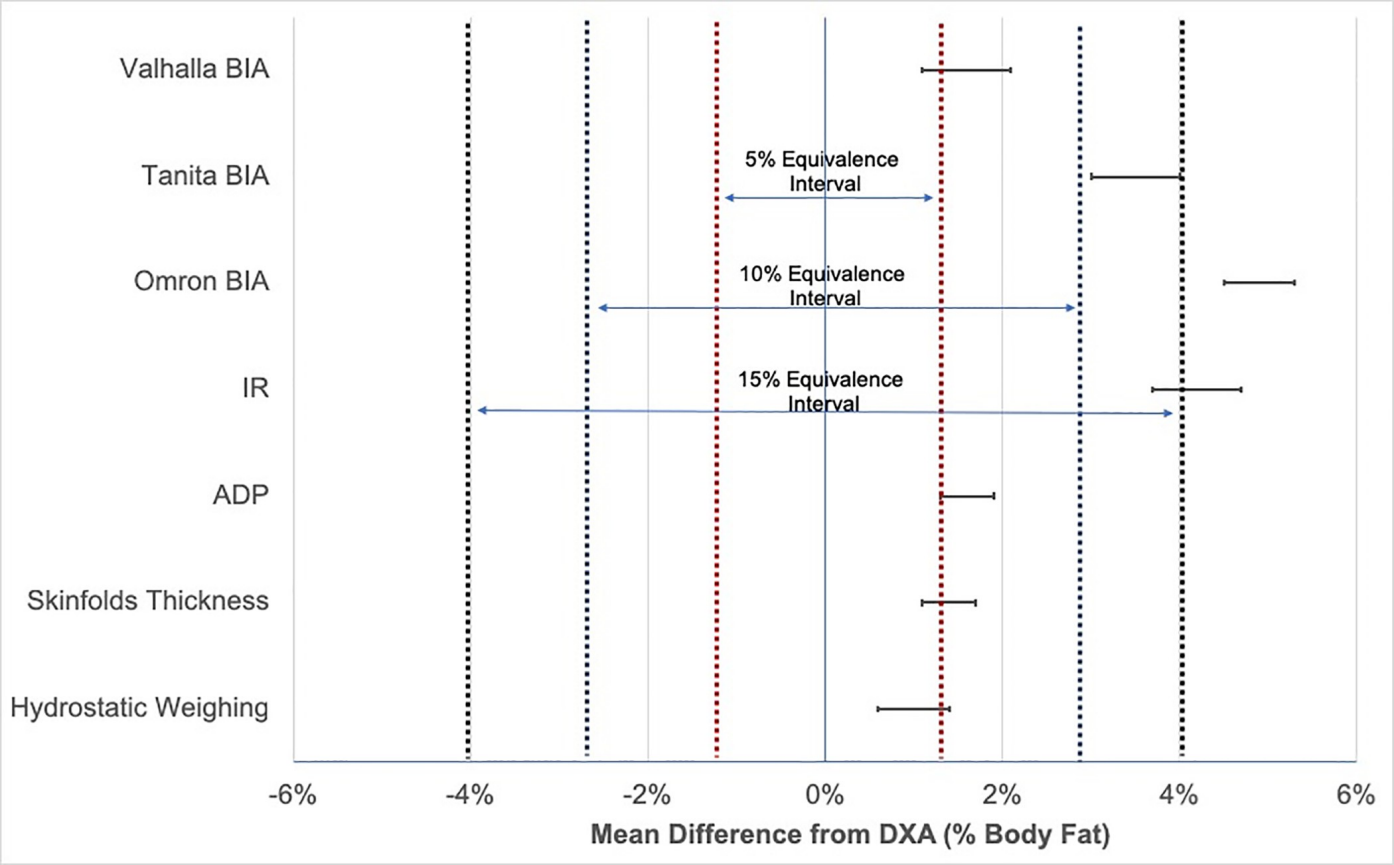
Error bar chart showing the relation of mean difference 90% confidence Intervals to various equivalence intervals. DXA stands for dual emission x-ray absorptiometry; BIA stands from bioelectrical impedance analysis; IR stands for near infrared reactance; ADP stands for air displacement plethysmography; x-axis is mean difference from percent body fat measured using hydrostatic weighing; upper and lower bounds of the Equivalence Intervals are denoted by dashed vertical lines; red dashed lines is the 5% Equivalence Interval; blue dashed lines is the 10% Equivalence Interval; black dashed lines is the 15% Equivalence Interval; Equivalence denoted by a respective 90% Confidence Interval falling within Equivalence Interval.

Table 3 presents the results of the employed equivalence testing using a 10% Equivalence Interval. The Equivalence Interval was set at +/- 10% of the criterion mean, which corresponded to +/- 2.7% body fat. Mean differences with DXA ranged from just 1% body fat using hydrostatic weighing to 4.9% body fat using Omron BIA. Rejection of the null hypothesis of non-equivalence was observed for four assessments: skinfolds thickness, ADP, 4-electrode Valhalla BIA, and hydrostatic weighing. The remaining assessments were determined non-equivalent compared to DXA, as the 90% Confidence Intervals did not fall entirely within the 10% Equivalence Interval. An error bar graph (Fig 1) is communicated to visually communicate the relation of 90% Confidence Intervals compared to various Equivalence Intervals obtained from DXA. Secondary analyses indicated that no surrogate assessment was statistically equivalent with DXA using a 5% Equivalence Interval and all surrogate assessments, except for Omron BIA and IR, were statistically equivalent with DXA using a 15% Equivalence Interval. Agreement with DXA at the individual level was quantified using MAPE (Table 3). MAPE ranged from 11.7% using skinfold thickness to 21.9% using the Omron BIA.

**Table 3:** Agreement in percent body fat estimates compared to dual-emission X-ray absorptiometry.

Assessment	Mean Difference (DXA–Surrogate; % Body Fat)	90% C.I. Mean Difference (% Body Fat)	10% Equivalence Interval (% Body Fat)	MAPE
Hydrostatic Weighing	**1.0%**^**†**^	0.6% - 1.4%	-2.7% - 2.7%	13.4%
Skinfold Thickness	**1.4%**^†^	1.1% - 1.7%	-2.7% - 2.7%	11.7%
ADP	**1.6%**^**†**^	1.3% - 1.9%	-2.7% - 2.7%	14.5%
IR	4.2%	3.7% - 4.7%	-2.7% - 2.7%	18.7%
Omron BIA	4.9%	4.5% - 5.3%	-2.7% - 2.7%	21.9%
Tanita BIA	3.5%	3.0% - 4.0%	-2.7% - 2.7%	17.2%
Valhalla BIA	**1.6%**^**†**^	1.1% - 2.1%	-2.7% - 2.7%	17.0%

*Note*: Criterion is percent body fat measured from dual-emission x-ray absorptiometry; 90% C.I. stands for the 90% Confidence Interval; Equivalence is denoted if the 90% C.I. falls completely within the Equivalence Interval

bold and † denotes statistical significance, *p* < 0.05

mean difference statistical significance denotes the null hypothesis rejection of non-equivalence; MAPE stands for mean absolute percent error; ADP stands for air displacement plethysmography; IR stands for near infrared reactance; BIA stands from bioelectrical impedance analysis; DXA stands for dual emission x-ray absorptiometry.

**Fig 2:**
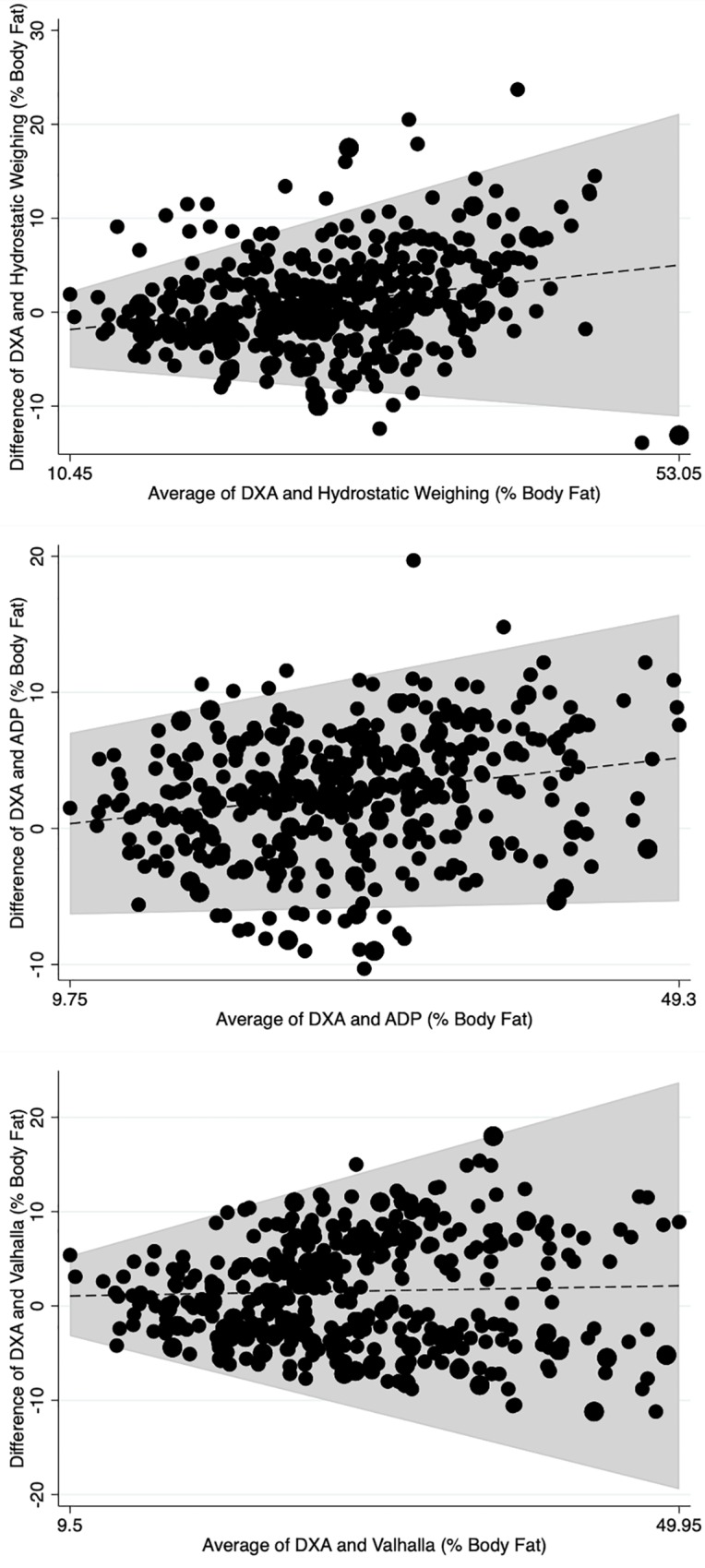
Bland-Altman Plots showing individual-level agreement between each lab surrogate assessment and percent body fat measured using dual emission x-ray absorptiometry. DXA stands for dual emission x-ray absorptiometry; ADP stands for air displacement plethysmography; shaded area is the 95% Limits of Agreement adjusted for heteroscedasticity across the range of mean values.

**Fig 3:**
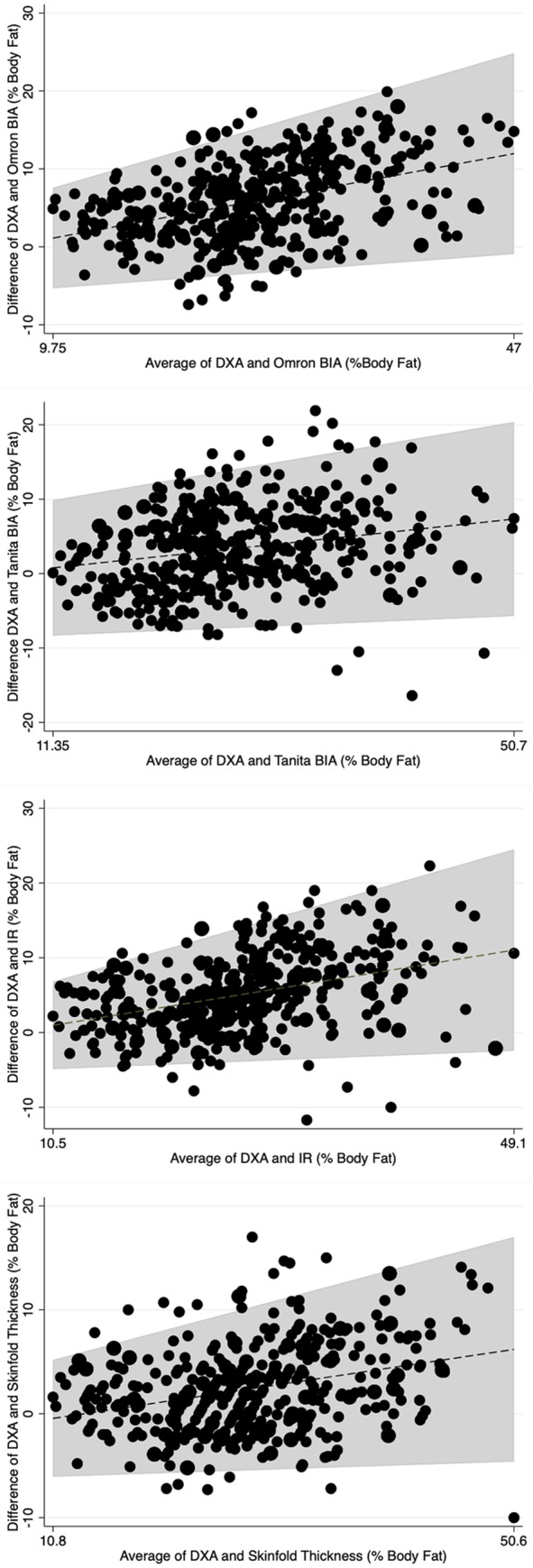
Bland-Altman Plots showing individual-level agreement between each field surrogate assessment and percent body fat measured using dual emission x-ray absorptiometry. DXA stands for dual emission x-ray absorptiometry; BIA stands for bioelectrical impedance analysis; IR stands for near infrared reactance; shaded area is the 95% Limits of Agreement adjusted for heteroscedasticity across the range of mean values.

Using Bland-Altman plots, there was evidence for heteroscedasticity when comparing DXA with all surrogate assessments, as there was a correlation between the mean differences and mean scores and the 95% Limits of Agreement were not homogenous across the range of values. Table 4 displays the correlation coefficients between mean differences and mean scores. Fig 2 and Fig 3 visually display the Bland-Altman Plots for each lab and field surrogate assessment against DXA, respectively. The grey shaded area is the 95% Limits of Agreement adjusted for heteroscedasticity. As seen by the varying 95% Limits of Agreement, differences between DXA and each surrogate assessment increased as mean percent body fat increased.

**Table 4:** Correlation coefficients between mean differences and mean scores for each surrogate assessment against dual emission X-ray absorptiometry.

Assessment	Pearson *r*	*p*-value
Hydrostatic Weighing	**0.23**^**†**^	< 0.001
Skinfold Thickness	**0.25**^**†**^	< 0.001
ADP	**0.25**^**†**^	< 0.001
IR	**0.33**^**†**^	< 0.001
Omron BIA	**0.39**^**†**^	< 0.001
Tanita BIA	**0.28**^**†**^	< 0.001
Valhalla BIA	0.04	0.784

*Note*: ADP stands for air displacement plethysmography; IR stands for near infrared reactance; BIA stands for bioelectrical impedance analysis

bold and ^†^ indicates statistical significance.

## Discussion

The purpose of this study was to examine the agreement in percent body fat estimates among 7 lab and field assessments in a sample of college aged students using equivalence testing. The results support that skinfolds thickness, ADP, 4-electrode Valhalla BIA, and hydrostatic weighing yielded statistically equivalent estimates in body fat using a 10% Equivalence Interval relative to DXA. Previous studies have assessed body composition using a limited number of assessments and the general linear model to assess group level agreement. The results support the use of specific lab and field assessments; specifically, the lab assessments of ADP, 4-electrode BIA (Valhalla), hydrostatic weighing, and the skinfolds thickness field assessment. MAPE ranged between 10%-20% across most assessments, however there was evidence of heteroscedasticity across the range of mean percent body fat scores, suggesting greater individual error at higher levels of percent body fat.

Valid body composition assessment is of importance to both researchers and practitioners within the fields of nutrition, exercise science, and public health [39]. This is especially important when assessing individuals, where the popular index for assessing body composition, BMI, is characterized as having inherent limitations [40]. Of the surrogate lab assessments observed in the current study, 4-electrode BIA, ADP, and hydrostatic weighing were all determined to be statistically equivalent to percent body fat measured using DXA.

Even though hydrostatic weighing has been considered the criterion in body composition assessment [41], administering the test is cumbersome, requires significant lab space, needs a high degree of administer training, and places a high degree of burden on the participant due to having to be completely submerged underwater [42]. More practical lab assessments throughout recent decades have become alternatives to hydrostatic weighing, such as ADP and DXA. Both of these lab assessments do require some administer training and are monetarily expensive, but subject burden is somewhat attenuated [43]. Hydrostatic weighing’s error with DXA was 1%, which is similar in previous studies, however some studies do show greater bias [44]. ADP’s absolute error with DXA was at 1.6%, which is again similar compared to other studies using younger adult samples where error ranges from 2–3% body fat [45]. Valhalla 4-electrode BIA absolute error was at 1.6%, which was within the +/- 2.0% body fat observed in other studies compared to a reference method [32, 46].

Of the 4 field assessments examined, percent body fat estimated using skinfolds thickness yielded the lowest percent error and was statistically equivalent with DXA. Skinfolds thickness requires some administer training, but is characterized as having low participant burden and is inexpensive [47, 48]. Health-related fitness test batteries often use skinfolds thickness to assess body composition across a variety of populations [49]. MAPE scores were the smallest using skinfolds thickness with an absolute measurement error of 1.4% compared to DXA. The minimal error observed for skinfolds thickness could be further attenuated using quality assessment training, ensuring that the participant is hydrated and in a fasted state, and ensuring that the assessment precedes any vigorous physical activity, which can cause sub-cutaneous water retention variability that may alter measurement.

Despite the positive findings yielded using skinfolds thickness, other fields assessments including 2-electrode BIA and IR did not show statistical equivalence with DXA. Two-electrode BIA and IR are practical, carrying relative low participant burden and are relatively inexpensive [50]; however, large measurement error is often observed using commercial BIA field assessments, especially in individuals with excess adiposity [51–53]. Two-electrode BIA may be more practical for personal use compared to 4-electrode BIA [52], however tetrapolar BIA may be more accurate. BIA is a method that measures resistance of electrical current through the body [54]. From obtained resistance scores, total body water and fat-free mass can be calculated [55]. Despite BIA’s practicality, dehydration can lead to overestimation of percent body fat and overhydration can lead to underestimation of percent body fat [55]. There may also be error related to the specific Tanita and Omron brand algorithms used to estimate body fat. Improvements in BIA accuracy may manifest from further calibration studies against reference assessments [52]. IR also carries with it several sources of measurement error with questionable validity against reference methods [56, 57]. Because of the lack of statistical equivalence and large MAPE, use of 2-electrode BIA and IR to estimate body composition should be interpreted with caution.

Results of this study yield important practical implications. Only one of the 4 field assessments of body composition were determined to be statistically equivalent with DXA. Researchers and practitioners should therefore use caution when employing these devices. Despite ease of use, absolute and relative error using BIA and IR may be practically significant. Because of the non-equivalence and relatively larger MAPE scores, assessment of individual health status based off of these field assessments is precluded. However, it is encouraging the degree of agreement of skinfolds thickness compared to DXA. The use of skinfolds thickness may be preferable over lab-based assessments because of lower subject burden and lower cost of performing the assessment. Therefore, within clinics where resources are limited, the use of skinfolds thickness performed by a trained technician may be very cost-effective.

There are several strengths to this study including the use of a relatively large sample of college students and the use of several lab and field assessments of body composition. The salient strength and novelty of the study was the use of equivalence testing. Additionally, we compared agreement across a range of equivalence intervals. Given these strengths however, there are limitations. First, the sample included college-aged students located from one university located in the western US; therefore, the results do not generalize to younger or older age groups. Second, college students were enrolled in exercise science courses; therefore, most of the participants had a good level of health-related fitness. Third, to maintain a relatively large sample size, results were not stratified by sex. Fourth, hydration status was not completely controlled for, which may decrease the validity of the BIA scores. Fifth, the observed results may have varied by body composition status (e.g., overweight/obese), this should be a focus for future research. Finally, all assessments do not measure percent body fat directly but utilize prediction algorithms. Results may have differed if other prediction equations were used across each of the assessments.

## Conclusions

In conclusion, the results support that skinfolds thickness, ADP, 4-electrode Valhalla BIA, and hydrostatic weighing yielded statistical equivalence in estimates of percent body fat relative to DXA. The results support the use the lab assessments of ADP, 4-electrode BIA (Valhalla), hydrostatic weighing, and the skinfolds thickness field assessment. Given the results from the current study, these assessments were the most valid body composition assessments in college-aged participants. Researchers and practitioners should use caution when assessing body composition and subsequent health risk when using 2-electrode BIA and IR field assessments. Valid assessments of body composition are essential for classifying health risk in any population. The current study provides important information on the relative and absolute agreement of various lab and field assessments that can be used in both researchers and clinicians.

## Supporting information

S1 Data De-identified data file. (CSV)
